# Women farm what they can manage: How time constraints affect the quantity and quality of labor for married women’s agricultural production in southwestern Nigeria

**DOI:** 10.1016/j.worlddev.2021.105800

**Published:** 2022-04

**Authors:** Rachael S. Pierotti, Sophia Friedson-Ridenour, Olubukola Olayiwola

**Affiliations:** a World Bank, Washington, DC, USA; b University of South Florida, Tampa, FL, USA

**Keywords:** Gender, Agricultural labor, Intrahousehold, Time use

## Abstract

Across sub-Saharan Africa smallholder farmers depend heavily on manual labor supplied by their households, families, and communities. Gender differences in the ability of farm managers to acquire needed labor has been linked with women’s disadvantage in agricultural productivity. This in-depth qualitative research in southwestern Nigeria builds on studies that document gender gaps by examining how men and women make sense of the allocation of labor within their households. Insights from observation over the course of one year and interviews with 93 participants are combined with evidence from existing literature to develop a framework that illustrates the conceptual links between constraints on women’s time use and the quantity and quality of labor available for their agricultural activities. We find that women’s time and labor constraints are rooted in common social expectations that men’s farm plots take priority and that a woman should only farm what she can manage without interfering with the agricultural production managed by her husband. Practically, this means that women’s household responsibilities and off-farm work limit their own farm labor and their ability to supervise hired labor. The prioritization of men’s plots also means that labor is allocated to men’s plots first in the day, which results in less labor and potentially less productive labor available for women’s farms. Also, women’s access to labor is especially constrained by seasonal fluctuations in labor demand because of the precedence given to men’s agricultural production. The conceptual framework is meant as a tool to be used in future research on time use, agricultural labor, and gender differences in agricultural productivity. It highlights the ways in which intrahousehold negotiations over labor and time use are not just about maximizing efficiency or productivity, but also about maintaining social hierarchies, roles, and responsibilities.

## Highlights

• A conceptual framework linking constraints on women’s time and the quantity and quality of labor available for their farms.
• Patterns of women’s time use are rooted in common expectations regarding gender roles and responsibilities.
• Time constraints limit women’s earnings from farming and make availability of money a determinant of what women can farm.
• Intrahousehold negotiations over labor and time use are about securing livelihoods and reinforcing social relationships.
• The conceptual framework is a tool for research on time use and gender differences in agricultural labor and productivity.

## 1 Introduction

Across sub-Saharan Africa agriculture is central to people’s livelihoods and food security (World Bank et al., 2009). Women account for an average of 40 percent of labor in crop production (Palacios-Lopez et al., 2017), but resource and opportunity constraints prevent them from optimizing their time (FAO, 2011). Women’s disadvantaged access to agricultural labor, both in terms of labor quantity and quality, constrains the productivity of the farms they manage. A recent study in six sub-Saharan African countries estimates a gender gap in agricultural productivity of between 13 and 25 percent, depending on the country and the crop (World Bank & ONE Campaign, 2014). The report identifies differences in access to agricultural labor as one of the main barriers to achieving gender equality in productivity.

Agriculture in Nigeria, as across sub-Saharan Africa, depends heavily on manual labor supplied by farmers’ households, families, and communities (Oluyole et al., 2013). A study in Nigeria found that women in the south and southeast of the country have lower levels of access to inputs than men, and productivity differentials were partially attributed to female plot managers using less male family labor (Oseni et al., 2015)^1^. These findings are echoed in research from other African contexts. Recent data from Tanzania, Malawi, and Ethiopia, show that limited access to male family labor on female plots accounts for a substantial amount of the productivity gap in agriculture: 97.3 percent in Tanzania, 45.2 percent in Malawi, and 43.7 percent in Ethiopia (Rodgers, 2018).

This in-depth qualitative study examined the taken-for-granted ways of organizing daily life that undergird observed gender gaps in agricultural labor. Over the course of one year, the research team iterated between data collection and analysis, conducting eight trips to three study communities in the southern state of Oyo, Nigeria. Observation of daily life was combined with interview data from 47 women and 46 men. The research focuses specifically on the labor constraints of married women in male headed households, the most common household structure for Nigerian women^2^. Married women often manage separate farm plots in addition to working on joint plots managed by their husbands. Women generally control the outputs from their own farm plots, and while they benefit from the plots managed by their husband, they do not control the outputs.

Data indicate that there are five primary types of time constraints that structure women’s daily life and influence their access to labor: 1) caretaking and household responsibilities, 2) off-farm income generating activities, 3) timing in the day for agricultural work, 4) dry versus wet season demands, and 5) short-term labor bottlenecks. These constraints both pull women’s time away from their farm plots and structure their time on them. They also affect the quantity and quality of household and hired labor on their farms. Furthermore, the data illustrate that these time constraints are rooted in common social expectations that men’s farm plots should take priority and that a woman should farm only what she can manage without interfering with the agricultural production managed by her husband. As a result, women have disadvantaged access to household labor and need cash to pay hired laborers. Thus, time constraints hinder women’s ability to earn money from farming, and also make the availability of money a key determinant of what women can farm.

The main contribution of this paper is the development of a conceptual framework to guide research related to gender differences in time use and agricultural labor. As such, it responds to calls for studies to take a more analytic approach to understanding time allocation (Seymour et al., 2020, Stevano et al., 2019). Studies from a variety of contexts in sub-Saharan Africa find that, on average, women work more total hours than men and are more likely to be time poor (Blackden & Wodon, 2006). The importance of these time use studies is confirmed by research that finds that women’s empowerment is undermined by excessive workloads (Malapit et al., 2019).

This study builds on time use research by examining the social dynamics that produce and reproduce gender gaps in agricultural labor, and incorporates insights from research that demonstrates the entwined nature of how productive and reproductive work are organized in everyday life (Stevano, 2021). We illustrate how women’s agricultural production is embedded in a complicated intrahousehold negotiation over time, which impacts the quantity and quality of women’s labor on their own farms, and also their ability to mobilize other labor. As such, this study furthers the insight from recent feminist political economy research that shows how the imperatives of social reproduction affect the organization of production (Stevano, 2021, Mezzadri and Majumder, 2020). Social reproduction is comprised of “all those activities involved in the production of life,” including biological reproduction, care work, production of home goods and services, maintenance of intimate relationships, and (re)creation of community social structures and ties (Elias and Rai, 2019:203). The (gendered) way that this labor is distributed across the population has important implications for the organization of productive work.

While the particular types of time constraints presented in the conceptual framework are specific to the communities where this research was conducted, the relationship between time-related constraints and labor can be used as a roadmap of potential interrelated pathways to explore when trying to understand gender gaps in agricultural productivity in other contexts. Moreover, the framework provides a tangible illustration of the importance of examining underlying social logics, or taken-for-granted ways of organizing productive and reproductive work, when trying to understand why women have differential access to agricultural labor and hence lower productivity.

### 1.1 Time constraints and agricultural labor

Time use studies are one important source of information about labor allocation within households, and they have shown that women face a variety of time-related constraints. One of the most documented time constraints women face is the greater amount of time they spend than men on domestic tasks including childcare, food preparation, and other household responsibilities (FAO, 2011). Advances in time use surveys that allow for the documentation of multitasking, especially the combination of childcare with other forms of work, have further improved our understanding of women’s work burden (Malapit et al., 2019). In some contexts, women who have children allocate even less time to wage work and more time to unpaid domestic work than women without children, indicating that lifecycle factors are important to understanding time use patterns and labor availability (Irani and Vemireddy, 2021).

In addition to fluctuations in response to lifecycle and other domestic responsibilities, women’s time use also changes throughout the agricultural season, in ways that are different from the seasonal variation of men’s time use (Wodon and Beegle, 2006). A study from southwest Nigeria, for example, found that men have more leisure time during dry season, while women have more leisure time during rainy season (Adeyonu, 2012). Time use surveys often fail to capture this kind of seasonal variation, which can risk missing important differences in labor allocation between men and women (Lentz et al., 2019, Stevano et al., 2019).

Time constraints affect not only the quantity of labor available for farming, but also the quality of that labor. To date, time use data have been limited in their ability to capture labor quality (Gollin, 2018). Time use surveys often ask farmers to recall labor for the previous season, but as Doss (2018) points out, recall surveys generally take a day of work as a fixed input, without taking into consideration variation in hours worked or level of effort. Labor quality may vary, for example, by type of laborer and type of farm (i.e. female vs. male owned). Labor performed by plot managers and/ or household members might be of higher quality than work done by hired laborers. Hired labor requires supervision to ensure quality (Feder, 1985), and women who are time constrained often lack the needed time to monitor labor. Women also face potential challenges with the quality of family labor on their farms. A study from Malawi found lower returns to male labor on female-managed plots than male-managed plots (Kilic et al., 2015).

The quality or value of labor to agricultural production also depends on whether it is available when needed. Agricultural labor performed at sub-optimal times of the season can result in yield reductions. Many agricultural tasks, including land preparation and planting, are time-sensitive, and when performed at the right time can be much more effective than when done after the optimal-window has passed (Gollin, 2018: 14). Collectively these studies highlight the need to combine insights from time use studies with studies of agricultural productivity to examine gender differences not only in the number of hours of labor, but also in the value of that labor for agricultural production.

### 1.2 Intrahousehold negotiation and agricultural labor

For women farming in male-headed households, the amount and quality of labor that they can access for their farm plots is, in part, a function of intrahousehold negotiations. Decades of research from a variety of contexts in sub-Saharan Africa have found that households do not collectively maximize agricultural production as one unit. Udry (1996), for example, finds that in Burkina Faso output on women’s plots is about 20 percent lower than output on men’s plots within the same household, planting the same crop in the same year. These gendered productivity differentials are attributed to differences in the application of fertilizer and also importantly, labor (i.e. household male labor, children’s labor, and non-household labor are all used more intensively on men’s plots). This model of the household, where there are both joint and separate plots, has been documented across much of sub-Saharan Africa, including in Nigeria (Ezumah and Di Domenico, 1995, Doss and Quisumbing, 2020).

Research on gender differences in agricultural productivity draws primarily on collective or noncooperative bargaining models of the household in which resources are not pooled and individuals make separate decisions about consumption and investment (Doss and Quisumbing, 2020). In this model, the “conjugal contract” is important to men’s claims to women’s labor on their farms, and likewise women’s claims (albeit more limited) to men’s labor on theirs (Doss, 1996: 19-21). Importantly, noncooperative bargaining models allow for a nuanced understanding of people’s behavior within households; people cooperate in some areas, but not in others (Doss and Meinzen-Dick, 2015). In noncooperative households, people do not necessarily make decisions that maximize household efficiency or productivity, but rather their decisions are also motivated by the desire to uphold dominant social hierarchies, even when this comes at an economic cost (see, for example, McPeak and Doss, 2006). Household members may also accept inequality in the allocation of productive resources if it means access to shared resources in times of need (O'Laughlin, 2007).

The noncooperative model of the household is particularly relevant to this study where many women farm jointly with their husband on larger family farms, from which they benefit but do not control the outputs, and also manage a smaller plot of their own, from which they do control the outputs. Outputs from women’s farms often contribute to the maintenance of the shared household unit; women not only spend their money on private goods like clothing for themselves, transfers to their natal family, and social obligations, but also on shared household needs like investments in children’s education and smoothing household consumption. Thus, in this context, decisions about agricultural labor within households should be understood as part of a complicated negotiation of both cooperation and competition over time and resources with benefits accruing to individuals in some instances, and the household in others. The outcomes of these negotiations are mediated by economic interests, but also social expectations.

In this study we focused on intrahousehold negotiations, concentrating on the social relations visible within the time and space of the data collection activities. This focus is not meant to deny the importance of broader patterns of political economic investment and exclusion in Nigeria, which also have critical implications for the livelihood strategies of all households (Berry, 1993). For example, arable land and off-farm income generating activities are not equally accessible to all, leading to social differentiation among smallholder farmers, as in other contexts (e.g. Cousins et al., 2018). Additional research would complement this study by examining the micro–macro connections between, on the one hand, how households negotiate and organize production and reproduction, and, on the other hand, structural forces that shape the labor market and the role of cash for meeting household needs (Stevano, 2021).

Recent survey research has advanced our knowledge of average gender differences in time use, but there is a need for greater understanding of how people make sense of labor allocations. This paper, therefore, starts to fill a gap in this literature by building a conceptual framework that: 1) Clearly demonstrates how social expectations undergird women’s time constraints in agriculture, 2) identifies five specific types of time constraints faced by women farmers, and 3) shows how these constraints relate to the quantity and quality of manager, household, and hired labor available on women’s farms.

## 2 Context

Qualitative data were collected in three study communities in the Ido Government Area in Oyo State, Nigeria. All study communities are in the rainforest agro-ecological zone. The primary crops cultivated include maize, tomatoes, jute leave, cassava, cocoyam, plantain, banana, beans, rice, vegetable leaves, and pepper. Cocoa is also cultivated by indigenes who have inherited cocoa farms. Generally, women and men cultivate all crops. However, men dominate pepper cultivation, particularly in dry season. On average in these communities, men cultivate between five to ten acres of land while women cultivate between two “*igba*” (400 ridges) to three acres.^3^ There are both monogamous and polygynous households in the study communities. Both types of households often include extended family members who eat from the same pot. In addition to farming, men and women in the study communities are engaged in off-farm income generating activities, but it is more common for women to perform this kind of work than men. Women process agricultural products (making fufu, frying garri, extracting palm oil), do petty trading, and hair dressing.

Key informants identified potential study communities that are sources of produce sold at a central market frequented by wholesalers and retailers from the Ibadan metropolitan area. Nineteen communities were identified, from which three were selected. The selected communities stood out because of the heterogeneity of their populations which included people from various ethnic groups including Yoruba, Igbo, Idoma, Egede, Igala, Hausa (Nigeria), and other migrant populations such as Ahori and Dendi (Benin). The first two communities, Aba-Alabi and Aba-Dejo^4^ were smaller and closer to the central market, while Aba-Adigun was larger and further away. A census was completed in each of the study communities to collect basic information about the number and types of households (see Table 1 below).

**Table 1:** Household types.

Categories of Household	Number of Households: Aba-Alabi	Number of Households: Aba-Dejo	Number of Households: Aba-Adigun
Male-headed household cultivating only joint farm plots managed by men (one manager)	10	18	87
Male-headed household cultivating both joint and separate farm plots (two manager)	30	20	63
Female-headed household cultivating farm plots	2	1	10
Male-headed household not cultivating farm plots	N/A	N/A	8
Female-headed household not cultivating farm plots	N/A	N/A	4
Total number of households counted	42	39	172

### 2.1 Description of study communities

Aba-Alabi is about 7.8 km from the main market in the area, approximately a 90-minute walk. Aba-Dejo is about 5kms from the main market, approximately a 60-minute walk. The distance between Aba-Alabi and Aba-Dejo is a 25-minute walk. Given their proximity to one another, they share some services including a community health center and a primary school. Between the villages are farm plots where women from both villages cultivate vegetables. For the purpose of this study, Aba-Alabi and Aba-Dejo were combined and treated as one site because they share infrastructure and farmland. Aba-Adigun is bigger than the other two villages. It is about 17.1 km from the main market in the area, more than a three-hour walk. The village has a road (graded but not tarred) that runs through it, which connects to the major road to Ibadan.

### 2.2 Household types

There are two primary types of farming arrangements within the households in the study sample, as identified by the study participants themselves: one manager and two manager households. In one manager households, the husband and wife cultivate farm plots together and the husband is viewed as the manager. It is expected that money earned from jointly-farmed plots is controlled by the husband but will benefit the household. Research participants referred to plots managed by men as the “husband’s farm” even though women contributed labor. In two-manager households, in addition to joint plots, the wife also cultivates a separate plot and maintains control over the earnings from it. In two-manager households, where the wife cultivates separately, the husband still performs some labor on her plot. These categories of household type shift over time and should be understood as Weberian ideal types that are never perfectly reflected in the messy reality of everyday life. Women might cultivate separately one season and not the next, for example, and household composition is not stable over time. Nonetheless, the distinction between these two ways of organizing household production—one manager versus two-manager—was meaningful to study participants who expressed strong opinions about whether it was good for women to manage their own plots. Household categorizations that appear in Table 1 represent the primary way in which respondents identified and/or researchers observed farming within the household at the time of research. The analysis presented here is based primarily on women’s experiences living in male headed households with either one or two managers.

### 2.3 Labor types

There are five major types of labor used by farmers in this study: self-labor (manager labor), household labor (spouse, children, other family members), “*aro*” (cooperative labor), daily paid labor, and yearly paid labor. Manager and household labor accounted for the majority of labor performed on all plot types. The cost of daily paid labor varies depending on the nature, quantity, and timing of tasks undertaken. Payments are most often made in cash and managers are expected to either feed laborers or pay for their food while they work. Yearly paid laborers live with the manager/master for a period of one year (January to December) at an agreed price (between ₦180,000 and ₦250,000), which is usually paid at the end of the year. Food, housing, and other living expenses are covered in addition to the lump sum payment. These laborers are usually migrants and are almost exclusively employed by men. Women hire yearly laborers to do day jobs once laborers have completed tasks for their masters and are released to work for themselves in the afternoons and on Sundays. *Aro* is an informal labor arrangement where a group of farm managers come together to work on one another’s farm plots on a rotational basis. While still practiced, *aro* was relatively uncommon, especially among women.

## 3 Methods

This research employed an iterative approach to data collection. We viewed each household as a case study, striving to understand the processes at play in each case, and comparing across cases to test emerging hypotheses (Small, 2009). All data were collected by the third author and one other Nigerian researcher. Research sites were visited a total of eight times between November 2018 and November 2019. Visits were spread throughout the year to capture labor patterns at different points in the farming cycle. The first few visits in November 2018 through January 2019 coincided with peak harvest of cocoyam, rice, plantain, and vegetables. Other ongoing labor activities included watering of vegetable farm plots and drying of harvested beans. During these visits, researchers sought permission for the study from village heads and conducted unstructured interviews to build rapport and collect basic information about farming practices and types of agricultural labor. Planting of cassava, maize, and cocoyam was ongoing during visits at the beginning of the rainy season in March, April, and May 2019. Data collection during those months consisted of semi-structured interviews focused on agricultural labor on the participants’ farm plots. Visits in August and September 2019 were toward the end of the rainy season and during harvest of some crops. Data collection again consisted primarily of semi-structured interviews with an updated interview guide that included new participatory activities to measure time use, detailed probes about labor allocation, and specific questions about growing pepper for sale. The last visit was during the dry season in November 2019 and included more semi-structured interviews based on a guide that had been further refined to elicit explanations of underlying social logics, in addition to details about labor allocation. Research participants were interviewed only once, although a few were recontacted for follow-up or clarification questions. The sample was comprised of new people at each visit, allowing the team to examine the relevance of previously identified patterns and logics of labor allocation in different households each time (Table 2).

**Table 2:** Number of interviews.

Study Site	Interviews with women	Interviews with men	Small group discussion with women	Small group discussion with men
Aba-Alabi & Aba-Dejo	17	12	2	4
Aba-Adigun	24	20	0	2

Between each visit, the study team conducted preliminary analysis of the data to identify emerging themes and outstanding questions. The preliminary analysis was used to direct subsequent rounds of data collection. In addition, we reviewed the composition of the sample and decided who to target for interviews in subsequent visits. Variation in land ownership status was ensured by including both indigenous Yoruba households and migrant households. Nearly all migrants were restricted to renting land, even if they had been resident in the area for decades. Through the rolling recruitment process, we also targeted households with both relatively smaller and larger land holdings (Table 3).

**Table 3:** Interview participant demographics.

Demographics	Number of Participants n = 93
*Age*	
<20 years	6
21–39 years	50
40–59 years	22
60 years & above	15
*Ethnicity*	
Cotonou^5^	10
Egede	22
Idoma	6
Igbo	7
Yoruba	48
*Gender*	
Men	46
Women	47
*Marital status*	
Married	83
Monogamous	48
Polygynous	35
Not married	8
Widowed	2
*Number of children*	
0	18
1–2	12
3–4	35
>5	28
*Religion*	
Christian	52
Muslim	40
Traditional	1

^5^Cotonou is used to represent ethnic groups from the Republic of Benin.

This prolonged engagement allowed the researchers time to seek consent and permission according to local customs and to establish rapport with community members. Because of their familiarity, study participants (both male and female) appeared to speak easily and often joked with researchers. While critical for collecting private information, the familiar rapport may have encouraged participants to try to please the researchers. To minimize this potential influence, interviews were structured around factual questions regarding observable farming practices, rather than seeking to elicit statements of belief. In other words, we asked people to explain what they do and why they do it that way, rather than asking for their opinion about how things *should* be. In addition, variation in the responses to the few opinion questions, such as whether it is good for women to farm separate plots, indicate that participants generally expressed themselves freely. Most often, interviews were conducted privately, but small group discussions sometimes occurred organically when the researcher was invited to join a group that had already gathered for work or leisure activities. These were generally groups of 2–3 people who knew each other well. All interviews were conducted in Yoruba or Nigerian Pidgin. On average, each interview and small group discussion lasted between 45 and 60 min.

### 3.1 Data analysis

All interviews were transcribed into English by the researchers who did the data collection. The transcripts were uploaded to Dedoose, a qualitative data analysis software. Data were coded three times. First, data were coded using eight descriptive codes and four analytic codes. A second cycle of coding included the addition of 16 child codes. After reviewing all first and second cycle coding, researchers developed a data matrix recording, for each participant, information related to different kinds of agricultural labor, decisions regarding whether and when women farm separate plots, control over income, and constraints to productivity. The third round of coding made evident the links between time constraints and labor quantity and quality, leading researchers to develop the conceptual framework presented in this paper.

## 4 Findings and conceptual framework

As described above, this in-depth qualitative study sought to better understand how smallholder farmers think about the allocation of labor to plots managed by men and women. We wanted to know what aspects of labor allocation were the subject of intrahousehold bargaining and which aspects were viewed as fixed and not something that could be negotiated (Agarwal, 1997). The data clearly indicate that the participants share common expectations about the social organization of households and agricultural production that influence how they allocate labor. For the remainder of the paper, we call these shared understandings “social logics.” Social logics are the taken-for-granted ways of being, of interacting with others, and of making sense of the world around us (sociologists and anthropologists might call these cultural schemas, e.g. Boutyline and Soter, 2021). Cultural theorists have shown that mental models—ideas about the way things are and the way they ought to be—inform how individuals behave, often on a subconscious level (DiMaggio, 1997). Recent research on gender differences in agricultural production increasingly acknowledges the importance of gender norms, for shaping the options available to male and female farmers (Hillenbrand & Miruka, 2019). Gender norms are defined as informal and socially enforced rules that define acceptable behavior for men and women. Research on norms at the individual level emphasizes that behavior is influenced by perceptions of what other people do (descriptive norms) and what other people think is appropriate (injunctive norms) (Legros & Cislaghi, 2020). Normative perceptions are an important part of the cognitive framework that influences people’s actions. In this paper, however, instead of discussing gender norms, we have chosen to use the broader category of social logics because the cognitive or ideational models that are evident in the data are not limited to individual perceptions of other people’s behavior and expectations. More generally, these social logics structure what research participants recognize as possible and the meaning and value that they assign to particular behaviors (see Edberg & Krieger, 2020 for a discussion of a more culturally embedded conception of social norms).

**Fig. 1:**
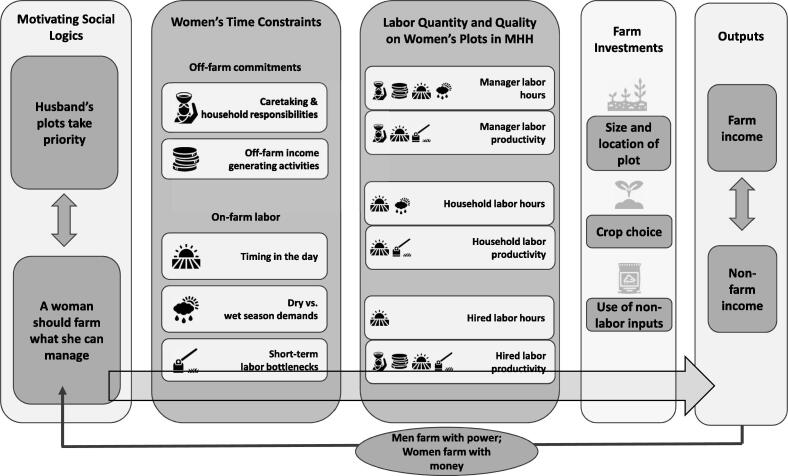
Conceptual framework: How time constraints affect the quantity and quality of labor for women’s farms.

Social logics subconsciously motivate behavior because people experience these logics as ubiquitous—it is just the way things are. Through a careful examination of how people explained the allocation of labor to men and women’s plots, we captured the social logics that underpin the observed time use and labor allocation patterns. While social logics can subconsciously motivate behavior, people also consciously draw on these logics as justification for action in response to requests for explanations of behavioral patterns (3Vaisey, 2009). Motivating social logics appear at the far left of the conceptual framework and are described first below (see Fig. 1).

Second, we describe five different types of time constraints that affect labor on women’s farm plots. Time constraints are grouped into those that pull women’s labor and attention away from their agricultural activities and those that structure the use of labor on the farm. We explain each of the time constraints, how the constraint is rooted in social logics, and how it affects the quantity and/or quality of labor for women’s agricultural activities.

The conceptual framework below is a graphical representation of these links, showing which time constraints affect which aspects of labor for women’s plots. There are different ways of measuring agricultural productivity, including productivity of land, labor, or total factor productivity (Doss, 2018, Gollin, 2018). For the purpose of this study, labor productivity was narrowly conceptualized as the effectiveness of labor to the farming enterprise, which is influenced by the quality of the laborers and the efficiency of the work performed (i.e. an hour of weeding at the right time is worth more than an hour of weeding at the wrong time). In the graphic we have indicated that the labor available for women’s farming has additional implications for other types of farm investments, such as crop choice, which in turn affects farm income. These links are apparent in some of the explanations provided by study participants, but they are beyond the primary focus of this study, which sought to explicitly examine gender differences in agricultural labor constraints.

In the final section of the description of study findings, we show that women’s time constraints lead to the expectation that women farm with money (hired labor) and men farm with power (manager and household labor). Women’s income, therefore, is central to their determination of how much farm they can manage.

### 4.1 Motivating social logics

Common explanations by study participants about gender differences in farming were twofold: 1) Men are the head of household and the family feeds from their farm, so men’s farms are prioritized and 2) Women farm what they can manage. The expectation that men’s farms would be prioritized was generally taken for granted and was readily accepted by both men and women. This had direct implications for the allocation of household labor. For example, Abdulrahman, a 37-year-old man explained,

*She [my wife] cannot cultivate as much as me because when I want to harvest my own farm produce she will need to assist me in doing that. So, her own farm plot is cultivated so that she will not just be there without anything and for her to be able to lay claim on something at least. That is why she is cultivating just a little.*

Another respondent, Jacob, echoed this sentiment. When asked why the labor goes to the farm plots managed by the husband first, he responded, “*Because the husband is the head of the family and he should be the one carrying all the responsibilities of the family*.”

Women expressed similar expectations about giving priority to men’s plots. When asked whose farm they go to more, their own farms or their husband’s, Serah and Hannah, both women in their 20s, stated that they go to their husband’s first. Serah explained, “*It is my husband’s own [farm that I go to first]. In fact, it is not all the time that I go to my own farm*.” Hannah added, “*If we want to go to our own, our husbands will say no…But we have money so that our farm will not spoil. We will give laborers money to go and do it for us*.” When further asked if they were okay with this arrangement, Hannah explained, “*We are happy. The reason why I said that we are happy is that when we harvest the farm produce, we are all eating it together*.” Women benefit from their labor on joint plots managed by their husbands, even if they do not directly control the output.

When asked about the size of women’s farm plots, both women and men responded that women farm as much as they can manage. As explained by study participants, because plots managed by men should take priority, women should farm only to the extent that their activities do not interfere with their husband’s agricultural production. Morufu, an 85-year-old man explained that the only reason why a husband would not allow his wife to expand her farm is if it will affect her work on his farm. When asked why her husband farms three acres while she only farms half an acre, Dupe, a woman in her 20s, answered, “*What I have the power to do is what I am doing…so that I will have time to go to my husband’s farm*.” Both men and women readily pointed to the importance of men’s farms as the reason why women farmed less, and this logic was central to assessments of what women could manage—women could farm using the labor left after men’s farms were taken care of.

These two social logics were nearly always referenced when explaining the intrahousehold allocation of agricultural labor, and these explanations appeared stable across different socio-economic groups including differences in groups by age, ethnicity, and migration status. This means that people used these mental frameworks to guide and explain their behavior. It does not, however, mean that labor patterns were fixed. In fact, there were exceptions to the norm that generally took one of two forms.

First, women withheld their labor from farm plots managed by their husband in protest when their husband was not fulfilling his responsibilities under the conjugal contract. Mourin, a younger woman, explained that she always goes to her husband’s farm to work when he asks unless they are fighting; *“[When we fight] I will not go [to his farm]*.” Fights might erupt over negotiations around household resources. One respondent, Saul, explained, “*When I get her [his wife] angry, or when she asks me to give her something and I don’t, maybe if I don’t have what she requests, she will decline [to come to my farm]*.” In other words, in rare instances, when men did not adequately provide for their wives or were perceived as withholding something, a wife might in turn withhold her labor on his farm as a form of protest. Under normal circumstances, however, women insisted that their labor was compulsory on the farm plots managed by their husbands.

The second exception to the prioritization of women’s labor on men’s farms occurred when women’s (off-farm) income was important enough to the livelihood of the household that they experienced less pressure to contribute labor to plots managed by their husband. This was true of Hassan’s wife. Hassan is in his late 50s, and when asked if his wife works on his farm he explained, “*If I want to plant on my farm, I don’t call [my wife]…I can employ laborers to help me plant. My wife is not usually available…The fufu work does not give her chance*.” His wife was able to prioritize work on her business most of the time because her husband recognized the value of her off-farm work. In rare cases, women even described paying for labor to take their place on their husband’s farm while they did off-farm work. Chineye, a younger woman in a polygynous marriage explained,

*[When] we [she and her co-wife] were doing cassava grain processing we didn’t have time to go to the farm plots. So, when they [laborers] help him to make ridges in his farm plots, we will give him money to look for daily paid laborers who will work for him. All the tasks we were supposed to do in his farm plots, the paid laborers will do it.*

While Chineye’s off-farm income gave her some latitude to negotiate her labor, freeing up her time still came at a cost. She and her co-wives were responsible for paying laborers to take their place in the fields. In the above cases, the constraints on women’s own labor were somewhat relaxed because of circumstances that weakened the prioritization of men’s farms, at least temporarily. These exceptions illuminate the dynamic relationship between production and social reproduction within households, and draw attention to moments of women’s agency and resistance in everyday life (Elias & Rai, 2019). The social logics frame the way that people think about labor allocation, but they are not behavioral prescriptions that operate uniformly across all situations.

These two social logics—that men’s farm activities should be prioritized and that women should farm what they can manage without detracting from the agricultural production managed by their husband—underpin the five time constraints faced by women farmers, and the sense people make of them. The time constraints are explained in the next sections.

### 4.2 Commitments that pull women’s labor away from the farm

The first set of time constraints relate to tasks that draw women’s labor off the farm, including caretaking and domestic responsibilities, as well as off-farm income generating activities. These off-farm activities limit the available hours a woman has to work on her farm. It should be noted that while both domestic responsibilities and off-farm income generating activities draw women’s labor off the farm, they are conceptually different. Domestic responsibilities are required for the continued functioning of the household, and there are strong social expectations that women will perform these social reproduction tasks as part of the conjugal contract. In contrast, while the money women make off-farm contributes to meeting household needs, if women fail to perform off-farm work there is not the same kind of social consequence as if they fail to care for their children or cook. In our conceptual framework, off-farm work is listed as a constraint because it is clear in our data that a range of social factors influence the tradeoff between off-farm and on-farm investment for women. These are discussed further below.

#### 4.2.1 Caretaking

Female respondents in this study, as across sub-Saharan Africa, spend significantly more time on reproductive work than men, reinforced by socio-cultural expectations around women’s domestic responsibilities. Benjamin, a 36-year-old man stated, “*The work of men is different from women’s own. We married them to be doing house chores and we are to work for them to eat on time*.” Expectations around women’s roles in the home limited their available hours to work on their farms, when in the day they were free to work, and led to multi-tasking, all of which had impacts on the quantity and quality of their labor.

Women with young children are particularly affected by caretaking responsibilities and men readily recognized that children limited women’s time to farm. Ganiyu, a 55-year-old man, explained that women who have young children farm smaller plots.

*You cannot compare it [a woman with young children] with a woman that is on her own that is no longer giving birth because…there is no child to disturb her…She will focus on her work…there are women that no longer have children in primary school…if she wants to do farming there is nothing like children again, except she cooks for her husband and that is all…you cannot even compare it to someone that has children to take care of.*

Caretaking responsibilities not only limited women’s availability to perform farm work, but also their ability to access markets and sell produce. When asked why she does not go to the market to sell produce herself, one respondent, Deborah, explained, “*I am carrying a baby. I cannot go because the market is not close…it is in the main city and they [the men] go in the middle of the night…we cannot be taking toddlers around during such hours*.”

The responsibility of women to care for children also led women to perform caretaking and farm work simultaneously, which has potential implications for the efficiency of their labor. Atanda, a 52-year-old man, recognized that as long as women are taking care of children their time and focus is divided; “*A woman that has a baby, her baby will be disturbing her and she will leave the farm work to attend to her child. All of these will be making her to be slow in her farm work. You know if I start working on the farm, nothing can hinder me*.” If they could, women used money to overcome these constraints. Rafat, a 35-year-old woman, explained, “*If I can’t go there [to the farm] by myself, I pay people to work in it for me…I use money to cultivate my farm plot…I have little children with me so there is no time*…*I hire labor*.”

While the caretaking demands of children limit women’s available time for farm labor, the financial responsibilities of having children are often, although not always, linked to women starting to farm their own plots. Eve, a 40-year-old woman, stated, “*Once a woman starts having children, she has to have her own farm. Is it all the time I will be asking him [my husband] for money?*” The responsibility for children increases women’s need for an independent source of income.

Women farming separate plots is, therefore, often directly intertwined with expectations around gendered caretaking roles and responsibilities which conceptually delimit, from the outset, the appropriate size and scope of their farms. Freeing up women’s time, therefore, won’t necessarily result in the increased allocation of that time to farm labor without also reimagining the role of women and women’s farms in the household. The life course stage can play an important role in determining the demands on women’s time. However, there is little research on the impact of life cycle issues on agricultural productivity (Peterman et al., 2010).

#### 4.2.2 Household responsibilities

Not only do women provide the primary caretaking for children, they are also responsible for a disproportionate share of household chores. Enitan, a 35-year-old woman, clearly stated,

*They [men] have more time than us…Women will want to cook food, wash clothes. I want to clean the house. But him, when he wakes up early in the morning and I have cooked for him, he prays then he is off to the farm. Maybe in the evening women will have time to go to the farm. That is why women’s power cannot be up to men’s power.*

As with the case of childcare, women’s household chores limit both women’s available labor for farming and the time of day she can farm. Her day is structured by the demands of the household first, whereas her husband’s time is primarily structured by the demands of the farm.

Men and women were both aware that domestic responsibilities limited women’s time to farm. Atanda stated,

*Her [his wife’s] power is not up to my own, let me tell you the major reason, I will leave the house in the morning around 7am, she will be home at that time preparing the food that we will eat. Before she comes around 9am to 9:30am, imagine the work I would have done….*

To compensate for time constraints, many women hire laborers to meet their labor needs. But hiring laborers presents other challenges. Because women are time constrained, they are often unable to provide appropriate supervision, which many explained is necessary to ensure laborers do good work. Olomid, noted challenges with labor quality;

*Let us say that [labor on] one acre is four thousand naira. Laborers will want to do two acres in a day to collect eight thousand naira, and they won’t do it the way the owner of the farm will do and will be rushing it so that he can do two plots so as to collect the money…The work the owner of the farm will do on the farm will be different from the laborers’ own.*

We investigated perceptions of whether women and men are equally respected as managers and whether they have equal ability to enforce labor contracts. We did not find convincing evidence of gender differentials (or gender equality) on either of those dimensions. Because reports of women’s competence as managers are heavily influenced by general assessments of women’s capabilities, these questions may be better answered with objective and systematic monitoring of contracts rather than qualitative interviews. Regardless, what we do see is that women are disadvantaged as labor managers because they have less time available for supervision.

In summary, women’s caretaking and household responsibilities have the potential to limit the number of hours that they can work, reduce the efficiency of their own labor hours because of distractions, and reduce the effectiveness of hired labor hours because of insufficient supervision.

#### 4.2.3 Off-farm activities

Many women (and men) in our sample have diversified income streams, although balancing multiple activities can be challenging. During a small group discussion, men were very clear, “*anyone that is doing business will not have the time to do farming…Farm work cannot be done with another work without you losing one…If you do two things, one will go bad*.” Likewise, Deborah explained, “*A business person cannot really focus on farming because she will go to the market whenever needs arise, so there is a limit to her being chanced*.” While men and women recognized that off-farm work would negatively impact on-farm work, it was common practice, and even encouraged, for women to farm and do business.

Across sub-Saharan Africa income diversification is common (Cousins et al., 2018; O’Laughlin, 1998). People allocate time and assets across various farm and off-farm activities to achieve a range of outcomes including income maximization, risk minimization, and income smoothing (Barrett et al., 2001). If women were free to allocate their productive labor hours however they choose, then it would not make sense to include off-farm work as a time constraint in our conceptual framework. But we posit that women are not entirely free in their decision-making about how to allocate their productive time. Data from study participants suggest multiple reasons why women’s labor hours may be pushed toward off-farm work, thus constraining their time available for farming.

While women often had multiple sources of income, many asserted that farming was the most lucrative. Bunmi, a 35-year-old woman, farms cassava, tomatoes, cocoa and has palm trees. In addition to farming, she is a trader. When asked what is most profitable, she explained, “*the one I am seeing more profit on is farming because when it starts growing, it is money*.” One explanation as to why women diversify is to smooth their income—farm income is too lumpy for their needs. A woman, Mourin, explained, “…*it takes a while before the farm produce will be ready for harvest and market. So, it is what we are selling that we use to manage ourselves.”*

Women might also diversify income because the barriers to increasing farm production are higher than the barriers to starting or expanding a business. As discussed above, household labor is prioritized on men’s farms first. This means that women have less labor to work on their farms, and less control over when that labor is available to them. The labor requirements for petty trading, or making and selling fufu, on the other hand, may be more flexible and more compatible with caretaking and domestic responsibilities. Thus, the structure of women’s lives and their responsibilities can encourage them to pursue off-farm income generating activities. Off-farm activities bring benefits, but it is not the case that women are able to make an unconstrained choice between off-farm and on-farm work.

It is also important to consider the way in which the underlying social narrative about farming as men’s work and business as women’s work might influence ideas about how women *should* spend their productive time. Precilia, a younger woman, was clear, “*It is men’s work [farming]. We women are doing it in a small scale…even the women that are doing it know that it is men’s work we are doing*.” A man, Ganiyu, voiced the opinion that, “*Assuming that Nigeria is good and everything is going the way it is meant to be, women will not be farming. They will be selling, and she does not need to trouble herself…Farm work is not women’s work*.” The data suggest that men are more willing to release their wives from doing labor on their plots if their wife is doing business than if she wants to expand her own farm. In other words, it was more socially acceptable for women’s productive hours to be spent doing business.

More research is needed to better understand the extent to which women are pushed or pulled into off-farm work (Van den Broeck and Kilic, 2018). It is not clear if women would choose to concentrate more time and energy on farming if they could leverage more labor. What is evident—as we illustrate in the conceptual framework—is that off-farm work, combined with caretaking and household responsibilities, impacts the availability of women’s time on their own farms and decreases their ability to supervise hired labor, which can have implications for labor quality.

### 4.3 Constraints that structure labor on the farm

The second cluster of time constraints have to do with sequencing of labor in the day, the season, and around short-term labor bottlenecks that structure when and how work is performed on women’s farms.

#### 4.3.1 Timing in the day

Both household labor and hired labor were primarily allocated to men’s farms during the first part of the day. People work for a longer period in the morning and the labor is generally more productive. Polina, a 20-year-old woman explained, “*When someone goes to farm in the morning time they work more than in the evening time. So, if my husband goes to his own farm plots in the morning, he will work better than when he comes to mine in the evening*.” Similarly, Theophilus, a 25-year-old man, stated, “*You can only do work for three hours in the evening time, but you can use six hours to seven hours to work in the morning*.” One woman, Rafat, explained that if both farms needed work in the same day, “*We will go to his [farm first]. For instance, he may go to his own farm plots in the morning time and go to mine in the evening time*.” It was common practice for all household labor to go to the farm plots managed by the husband first during the day, and those managed by the wife second. This arrangement was generally unquestioned.

Like manager and household labor, hired labor also tended to concentrate on women’s plots in the second half of the day. Women often hired yearly laborers to work on their farms in the afternoons and evenings. One man, Jude, has yearly laborers and explained, “*they [yearly laborers] are supposed to work from morning until 4 pm before they break… they may work on Monday, Tuesday, Wednesday, Thursday, Friday, Saturday. That is for the master*.” These laborers were free to do extra work for hire in the evenings and on Sundays, and this is when women usually hired them. Vivian, a 25-year-old woman, hired her husband’s yearly laborers to work on her plot: “…*we use them to work in our own farm plots too and they will come around evening*.” While men paid for the work of the yearly laborers on the household plots that they managed, women almost always had to pay separately for the work their husband’s laborers did on theirs.

Because all forms of labor are allocated to women’s plots second in the day, it is potentially less productive. In addition, because yearly laborers worked for women after completing work on men’s fields, and men had to release their laborers to do other work, women could not always count on labor being available when they needed it. In the conceptual framework, we have noted that constraints to which hours in the day are worked on women’s farms is linked to fewer overall hours of available labor and lower productivity for all kinds of laborers.

#### 4.3.2 Dry versus wet season demands

Labor demands ebbed and flowed with the seasons. In the study communities, the prioritization of women’s labor on men’s farms during dry season was most evident in the case of pepper, which required watering up to three times a day, a task primarily done by women. Respondents explained that pepper grown during the dry season was sold for a high price, but it required a lot of labor. The need for labor on men’s farms during dry season often resulted in women waiting to farm until rainy season.

One respondent, Hannah, described how she worked more on plots managed by her husband during the dry season, “*During the rainy season…there is nothing that I do for him other than to be weeding…During dry season, I will plant vegetables, plant tomatoes, corn and all of that*.” She further explained that she does her own farming starting in the fourth month. Peter, a 30-year-old man, explained, “*In the dry season, she’s [his wife] always at my place…she won’t [do anything in hers]…this is because the amount of work we do then won’t allow her to have time for hers until rainy season*.”

Women planted their own farms during the rainy season as a common response to the labor needs of men’s farms. And this aligned with the socio-cultural practice of prioritizing men’s farms. During a small group discussion one man, Israel, articulated the commonsense logic that women farm after labor demands are met on men’s farms;

*When I need her [my wife’s] help mostly it is when we need to water the pepper. That is at the early stage. So, she herself, she knows even where we come from the way they brought us up, it is after the husband’s farm before we make a woman’s farm plots. So, we have no option. Husband’s farm plot is the number one before we do a woman’s farm plot.*

Women farming in the rainy season had implications for the types of crops women chose, and they were not always the most lucrative crops. David explained that his wife cultivated vegetables that thrived during rainy season, but he was clear that the pepper he farmed during dry season was the most lucrative: “*[P]epper brings in income the most*.”

As noted in the conceptual framework, labor demands on men’s farms during dry season are linked to reduced manager and household labor availability for women’s farms. Seasonal labor demands are context specific and will vary by location. But, data from this study strongly support the general conclusion that labor allocation across men and women’s plots will fluctuate throughout the year (Adeyonu, 2012, Stone et al., 1995).

#### 4.3.3 Women’s plots tended second during short-term labor bottlenecks

Both men and women prioritized women’s time for men’s farms, and this also meant that labor was allocated to men’s plots first during short-term labor bottlenecks, which happened at various times throughout the season, and was particularly apparent during harvest time. Agai, a 30-year-old woman, explained,

*Once I know that my husband will harvest his jute and I also want to harvest my jute on the same day, is not easy to do the two together. So, I will leave my own for his own. Anytime my husband wants to harvest, the whole family will go but for mine is just me and my husband.*

Agai also explained that to help compensate for the loss of labor on her farm during task-specific labor bottlenecks, she hires labor: “*If it happens that I have grasses to clear on my farm and also my husband has [grass to clear], I will give my own to the laborers and I and my husband will do his own. We can’t do the whole work by ourselves*.” Using hired labor instead of manager or household labor, however, can come at a cost. Time constraints, driven by social expectations about prioritizing labor on the man’s farm, often prevented women from properly monitoring the hired labor, raising questions, as discussed previously, about the quality of work performed by laborers on women’s farms.

In the conceptual framework, time constraints related to short-term labor bottlenecks are linked with the productivity of manager, household, and hired labor. The productivity of manager and household labor can be reduced when tasks on women’s farms are performed at suboptimal times. The productivity of hired labor is affected by insufficient time for supervision.

### 4.4 Men farm with power; women farm with money

The sections above describe five different types of time constraints women face and the ways in which these constraints limit the quantity and quality of labor on their farms. While it was beyond the scope of this research to look at downstream outcomes, it was clear that time and labor constraints limited the amount of money women made farming. In the conceptual framework, women’s income links back to what they can manage because of the strong social expectation that what women can manage is a direct function of how much money they have to manage with.

There was a common assumption that men farm with power (i.e. their own strength complemented by the labor of other household members) and women farm with money (i.e. hired labor). Ibrahim, a 37-year-old man, for example, stated, “*Men use all their strength to work and women are using money to work…. where their [women’s] money stops is where their work stops*.” Similarly, Sukurat, a 40-year-old woman, explained that women can farm their own plots if they have the “capacity” to do so. When asked to clarify what she meant by capacity she said, “money.” We heard these explanations from both male and female respondents of all ages. Money allowed women to expand their own plots without disrupting their labor on their husband’s farm.

The importance of paid labor for women’s farms suggests an important dimension of inequality both between and within households. Households, and household members, with greater access to cash income generally have more options for overcoming time constraints (Johnston et al., 2018). Research on rural women’s livelihoods emphasizes how market forces have made the ability to live a good life dependent on access to cash income (“commodification”) (Stevano, 2021). Women with cash they control can hire labor for their farms, which is critical to their production given their restricted access to household labor.

Levels of wealth intersect with other dimensions of difference that also have implications for household and individual women’s livelihood strategies. For example, land ownership in the area is generally restricted to indigenous Yoruba men. This means they can plant perineal cash crops, such as cocoa, and can rent out land when they face labor shortages. Women in those households may have access to some of the cash generated from those activities. Their access to this resource, however, is dependent on their intrahousehold relationships, which means that men retain considerable control (Stevano, 2021). Women and migrants, therefore, have fewer options for responding to time and labor shortages for their farming activities.

When asked about constraints to farming, women often respond that insufficient money limits their production. But this is an incomplete answer. Women need money to farm because household labor is allocated first to the plots that men manage. The conceptual framework sheds light on the less visible constraints to women’s farming, showing that common social logics are linked to time and labor constraints that hinder women’s ability to earn money from farming, and also make the availability of money a key determinant of what women can farm.

## 5 Discussion and conclusion

The conceptual framework presented in this paper shows that women face multiple time-related constraints that draw labor off the farm and structure labor on it. It demonstrates the importance of measuring time use in ways that can capture the wide variety of time constraints experienced by female farmers. By mapping each time constraint to its associated impacts on labor hours and productivity, the conceptual framework illuminates that policies that alleviate one time constraint may not result in an equivalent increase in labor availability and productivity on women’s farms. Take for example the case of childcare. In the framework, caretaking maps to manager labor hours and manager and hired labor productivity (see Fig. 1). But, off-farm income generating activities, timing in the day, and dry versus wet season demands all also map to manager labor hours. So, addressing constraints around childcare, while important, may not be enough to increase manager labor hours as competing demands on her time may predominate. The multiplicity of constraints women face, and the ways they potentially interact, are important to consider when designing interventions aimed at increasing women’s agricultural productivity.

This framework, and the supporting data also highlight the importance of social logics in driving time use and labor allocation. Many of the time constraints women face are structured by social and cultural expectations around appropriate ways different people should spend their time. Opportunities to reallocate labor will be evaluated with reference to these social logics. Intrahousehold negotiations over labor are not just about maximizing efficiency or productivity, but also about maintaining social hierarchies, roles, and responsibilities.

At the same time, it is important to remember that while social logics can structure time use, these are not fixed realities, and time use is subject to negotiation. This research documented cases that deviated from the norm. For example, Chineye described how she paid for laborers to take her place on her husband’s farm, allowing her to prioritize her time for off-farm work. Cases like this indicate that some circumstances can provide openings for women to make different choices about their time. This is particularly true for women with considerable earnings from off-farm work. Economists tend to attribute livelihood diversification to efforts at income maximization, risk minimization, and income smoothing (Barrett et al., 2001). Feminist political economy research decries the compulsory “fragmentation” of working lives and the precariousness of the diversified livelihood strategies of those without access to substantial capital (Stevano, 2021). The findings of this study suggest a third perspective on rural women’s engagement in off-farm work: off-farm sources of cash income can influence intrahousehold negotiations over time and agricultural labor allocations.

Fruitful areas for further research include the examination of interhousehold heterogeneity in the way that time use is negotiated and the outcome of those negotiations. Relatedly, additional research is needed to examine how broader contextual factors—social institutions, market opportunities, and prevailing gender norms—affect negotiations over time and labor for women’s productive activities. Comparative research in other countries and other regions of Nigeria will help to illuminate these macro-micro linkages, and will situate the observed intrahousehold gender inequality in broader systems of socioeconomic differentiation (O'Laughlin, 2007). The conceptual framework is meant as a tool for design and analysis on future studies that examine time use, agricultural labor, and gender differences in agricultural productivity.

## CRediT authorship contribution statement

**Rachael S. Pierotti:** Conceptualization, Methodology, Supervision, Writing – original draft, Writing – review & editing. **Sophia Friedson-Ridenour:** Formal analysis, Writing – original draft, Writing – review & editing. **Olubukola Olayiwola:** Conceptualization, Methodology, Investigation, Data curation, Writing – review & editing.

## Declaration of Competing Interest

The authors declare that they have no known competing financial interests or personal relationships that could have appeared to influence the work reported in this paper.

## Footnotes

1 These analyses exclude households in southwest Nigeria, which was the region for this study.

2 As of 2015, only 14.6 percent of women in Nigeria live in female headed households (World Bank, n.d.).

3 According to respondents, one acre is approximately 15 *igba* or about 3,000 ridges.

4 Pseudonyms are used for all villages and study participants.
